# The impact of a regional training program on peripartum depression in territorial psychiatric services

**DOI:** 10.1192/j.eurpsy.2021.481

**Published:** 2021-08-13

**Authors:** F. Sceusa, A. Mauro, S. Pompili, L. Orsolini, U. Volpe

**Affiliations:** 1 Unit Of Clinical Psychiatry, Department Of Neurosciences/dimsc, Polytechnic University of Marche, Ancona, Italy; 2 Department Of Clinical Neurosciences/dimsc, School of Medicine, Unit of Psychiatry, Polytechnic University of Marche, Ancona, Italy

**Keywords:** Postpartum, women’s mental health, Perinatal Mental Health, pregnancy

## Abstract

**Introduction:**

The Unit of Clinical Psychiatry of the University Hospital “Ospedali Riuniti – Ancona”, in collaboration with the Marche Region Health System, is conducting a national observational project entitled “Measures related to the prevention, diagnosis, treatment and assistance of postpartum depressive syndrome”, aiming at promoting women’s Mental Health, particularly in pregnancy and peripartum period.

**Objectives:**

The primary objective is implementing all measures/interventions needed to promptly screening, early diagnosising, and supporting/caring women with mental health disease during pregnancy and peripartum period. A dedicated training program was performed by our clinical team belonging to the Peripartum Psychiatry Outpatient Service of the Unit of Clinical Psychiatry, at the University Hospital “Ospedali Riuniti”, Ancona, Italy, to a selected audience of Gynecologists/Obstetricians/Nurses/Psychologists/Psychiatrists/GPs and Pediatricians.

**Methods:**

The training program is a 2-days residential course, held on 21-22^th^ September, 2020. After the training program, all participants (n= 70) were asked to provide an informed consent and complete an online questionnaire to evaluate knowledge/opinions/experiences and clinical practices in the field of depression in pregnancy and postpartum.

**Results:**

A 40-items questionnaire investigated: a) general attitude in performing screening of depression/anxiety during pregnancy; b) overall knowledge about peripartum depression; c) overall knowledge about management/treatment; d) how physicians manage patients with peripartum depression/anxiety (i.e., how they perform screening/diagnosis/treatment during pregnancy, their levels of knowledge/confidence about psychopharmacology in pregnancy).

**Conclusions:**

The findings of the residential course may allow clinicians to adequately inform and help in drafting a preventive, screening and management program able to assist regional stakeholders in the prevention, diagnosis, treatment and assistance of perinatal depression.

**Disclosure:**

No significant relationships.

